# Reproductive Autonomy in Fertility Research in Sub‐Saharan Africa: A Scoping Review

**DOI:** 10.1111/sifp.70012

**Published:** 2025-05-05

**Authors:** Billie de Haas, Allen Kabagenyi, Syifasari Diennabila

## Abstract

Fertility research in sub‐Saharan Africa regularly indicates the need to increase women's reproductive autonomy. However, individual, female‐focused conceptualizations of reproductive autonomy tend to neglect the power dynamics both internal and external to couples and other intimate relationships that shape a woman's reproductive autonomy. Furthermore, they disregard the reproductive autonomy of men and other subpopulations and partners in intimate relationships. To identify gaps and evaluate the applicability of the concept, this scoping review clarifies how reproductive autonomy has been assessed and applied in fertility research in sub‐Saharan Africa. Eligible for inclusion were empirical peer‐reviewed publications, including quantitative, qualitative, and mixed‐methods research, published since 1994. Out of 1568 articles screened, 18 met the inclusion criteria. Most publications were quantitative in nature and focused mainly on the reproductive autonomy of women. Our key finding is that the reproductive autonomy of partners in intimate relationships, and of women in particular, is restricted at the community level in order to preserve the status quo of social power structures in society. In line with a reproductive justice approach, more research should focus on the reproductive autonomy of vulnerable and understudied populations as well as on the interpersonal and contextual dynamics that suppress reproductive autonomy in communal settings.

## INTRODUCTION

Reproductive autonomy is an essential prerequisite for people to achieve their desired fertility as well as other related reproductive health needs, such as access to a contraceptive method of one's choosing (Elwan and Raidoo 2020). Recent decades in sub‐Saharan Africa have shown a strong correlation between declining desired and realized fertility at the aggregate level, although there is variation between countries (Casterline and Agyei‐Mensah 2017). Despite observed trends, it can still mean at the individual level that people do not realize their ideal number of children under the conditions of their choice (Yeboah, Kwankye, and Frempong‐Ainguah 2021). For instance, a study in rural Northern Ghana found that people were having fewer children than desired because of the outmigration of spouses and environmental stress on livelihoods and as a strategy to cope with food insecurity (Adaawen 2015). In this context, the International Conference on Population and Development (ICPD) Programme of Action acknowledged in 1994 that reproductive autonomy is an important end in itself, rather than only a means to influence population change through population policies (Gietel‐Basten 2024; UNFPA 2014).

Reproductive autonomy has various definitions. The following definition closely relates to the ICPD 1994 Programme of Action formulation: reproductive autonomy is “the extent to which women and couples can determine freely whether and when they have children” (Potter et al. 2019). Both Upadhyay et al. (2014) and Purdy (2006) emphasize the role of power in their definitions of reproductive autonomy, that is, “the power to decide when, if at all, to have children; also, many—but not all—of the choices relevant to reproduction” (Purdy 2006) and “having the power to decide about and control matters associated with contraceptive use, pregnancy, and childbearing” (Upadhyay et al. 2014). These matters include not only the right to decide whether, when, with whom, and how to have children but also the right to make choices about one's body, sexual identity, and behaviors (Crawford et al. 2023). To increase reproductive autonomy, and that of women in particular, the ICPD 1994 Programme of Action stipulated the need for gender equality and access to health care and education (UNFPA 2014).

### Disentangling Reproductive Autonomy and Related Concepts

Since the adoption of the ICPD Programme of Action in 1994, many studies have been conducted to understand and explain people's, mostly married women's, reproductive opportunities, behavior, and outcomes (Edmeades et al. 2018; Lee‐Rife 2010). To conceptualize the power or freedom to decide on reproductive matters, studies tend to focus on the role of autonomy, empowerment, agency, coercion, control, and choice. These concepts are often used interchangeably, but, although they are related, their scope and meaning are not the same (Lee‐Rife 2010; Pratley 2016). In addition, the same concepts are defined differently (Osamor and Grady 2016). Therefore, it is important to understand how these concepts relate.

Similar to how empowerment, agency, and autonomy relate to each other, the dimension of reproductive empowerment is an overarching concept comprising reproductive agency, which again comprises the narrower concept of reproductive autonomy. Building on research by Eerdewijk et al. (2017), Kabeer (2001), and Malhotra and Schuler (2005), Edmeades et al. (2018) construe reproductive empowerment as a dynamic concept consisting of both reproductive agency and process. Reproductive empowerment can therefore be both an outcome and an ongoing process of change. This contrasts with the static concepts of reproductive agency and reproductive autonomy. Consequently, Edmeades et al. (2018) define reproductive empowerment as

both a transformative process and an outcome, whereby individuals expand their capacity to make informed decisions about their reproductive lives, amplify their ability to participate meaningfully in public and private discussions related to sexuality, reproductive health, and fertility, and act on their preferences to achieve desired reproductive outcomes, free from violence, retribution, or fear.

Previous literature has also considered the need for resources and changes external to individuals, such as gender norms within the sociocultural context, as enabling factors or catalysts for empowerment (Khader 2016; Kabeer 2001). However, Malhotra and Schuler (2005) argue that these are not features of empowerment itself because agency, as its component, requires that individuals themselves are the agents of change.

The concept of agency is considered to consist of the three components of choice, voice, and power. An agency is defined as “the capacity for purposive action that draws on social and material resources at multiple levels to realize preferences and choices, enhance voice, and increase power and influence” (Edmeades et al. 2018; Eerdewijk et al. 2017). Edmeades et al. (2018) distinguish three levels of agency: the individual level; immediate relational agency, for example, in interpersonal relationships; and distant relational agency, as with people in the community. The individual‐level agency is similar to how Yount, James‐Hawkins, and Abdul Rahim (2020) define intrinsic agency as “consciousness of one's capabilities, rights, and aspirations.” They also distinguish two other components of agency: instrumental agency, which is “the power to make one's own strategic life choices” and collective agency, which is the “identification of group goals and joint actions to pursue those goals” (Yount, James‐Hawkins, and Abdul Rahim 2020). Distinguishing collective agency means that empowerment and agency can exist both at the individual and group levels, whereas autonomy is an individual concept (Khader 2016).

Agency at the individual level (Edmeades et al. 2018) and intrinsic agency (Yount, James‐Hawkins, and Abdul Rahim 2020) are also similar to what Khader (2016) calls “thin relational autonomy” (TRA). TRA is “the capacity to act in ways consistent with one's values,” which requires the ability to form and reflect on values (Khader 2016). TRA is a type of personal autonomy, characterized by “the psychic capacity to shape one's self in a way consistent with one's values” and serving as an essential component of empowerment (Khader 2016).

As Khader (2016) explains, it is often assumed that an autonomous person is not influenced by relationships or context. In fact, Khader (2016) argues that TRA is very much a relational concept. Therefore, it facilitates a person to overcome oppressive relationships or social norms. As an internal capacity, TRA enables a person to know what they value. However, this does not automatically mean that they are also able to act upon it. Conversely, if a person has “instrumental agency” without TRA, they may have the ability to act, but within a limited range of perceived options or a restrictive environment. An example is covert contraceptive use, in which women are able to use contraception, but they do not feel they have the freedom to do so (Princewill, De Clercq, et al. 2017). Therefore, studying the narrower concept of autonomy, rather than the broader concepts of agency and empowerment, better exposes whether a person actually feels free from external oppression at the immediate and distant interpersonal and contextual levels to decide in accordance with their own values.

The concept of reproductive coercion underscores that reproductive autonomy is a relational and contextual concept. It focuses on forms of external oppression that could restrain reproductive autonomy at various levels, including interpersonal relations and in the sociocultural and legal context. Graham et al. (2023) define reproductive coercion as

the act of removing or limiting reproductive autonomy to control reproductive decision‐making freedoms and choices to hold power over reproductive autonomy. It occurs through socially and culturally embedded systematic control and oppression of reproductive rights, beliefs, conceptualisations of gender roles, behaviours, attitudes, and actions, practices, policy, law, and legislation resulting in gender inequality and other intersecting forms of oppression (particularly ability, ethnicity, and sexuality). This manifests and is experienced at the interpersonal level in multiple ways as an artefact of interconnected and interacting forces across the social, cultural, institutional systems and structures, organisations, and the state, which create the context within which reproductive coercion occurs.

The different concepts discussed have in common that they are concerned with the degree to which people can control and decide about their own (reproductive) lives, taking into account their various interpersonal relations and the context in which they live (Jejeebhoy 2000; Malhotra and Schuler 2005). The timing of measurement matters because people's perceived degree of control may fluctuate over the life course (Lee‐Rife 2010). Furthermore, these concepts are multidimensional. This means that reproductive autonomy, reproductive agency, reproductive coercion, and reproductive empowerment are dimensions of their respective overarching concepts, and each dimension may influence reproductive matters in a different way (Malhotra and Schuler 2005; Edmeades et al. 2018; Båge et al. 2024; Jejeebhoy 2000). For instance, people may have economic autonomy and simultaneously lack reproductive autonomy (Lee‐Rife 2010). For this reason, it matters whether a study measures the influence of autonomy as a whole or only reproductive autonomy on fertility and other reproductive outcomes.

Similarly, considering that autonomy measures relational power, a person may have reproductive autonomy in relation to their spouse and at the same time not have reproductive autonomy in relation to a relative or healthcare provider. This shows the importance of understanding the various interpersonal and contextual levels in which reproductive autonomy is measured or understood (Mandal et al. 2025; Malhotra and Schuler 2005). In the context of sub‐Saharan Africa, Mandal et al. (2025) indicate the significant role of male partners and healthcare workers in women's reproductive autonomy, thus emphasizing the importance of distinguishing between the immediate and distant relational components of reproductive autonomy in this context.

### Reproductive Autonomy of Different Populations in the Sub‐Saharan African Context

In line with the ICPD 1994 Programme of Action, studies on desired fertility in sub‐Saharan Africa regularly focus on women and the need to increase their empowerment, level of education, and access to contraception (D'Souza et al. 2022; Liu and Raftery 2020). However, such individual, female‐focused conceptualizations of reproductive autonomy may overlook the fact that reproductive reasoning and behaviors are not just a result of individual characteristics of women. Rather, reproductive reasoning and behaviors are highly contextual, and reproductive autonomy is a relational concept shaped by interpersonal and contextual power dynamics both internal and external to couples and other intimate relationships, such as polygamous relationships (see Figure 3) (Rich et al. 2021; Buser et al. 2023; Graham et al. 2023; Blanc 2001). Interpersonal power dynamics between intimate partners may concern partners’ communication about their fertility desires and the extent to which joint decision‐making is taking place (Stein, Willen, and Pavetic 2014). For instance, sociocultural norms may prompt male partners to have more say in the decision‐making, and, as a result, they may influence women's abortion trajectories or lead women to covert contraceptive use (Strong 2022; Sarnak and Gemmill 2022).

Acknowledging these interpersonal power dynamics, Upadhyay et al. (2014) developed a scale in 2014 to measure women's reproductive autonomy. The authors argued that research on women's reproductive health often focuses on their overall autonomy, rather than on reproductive autonomy specifically. Furthermore, they pointed out that studies often consider individual characteristics only, such as women's education and financial means, whereas a woman's reproductive autonomy is usually a result of the power she has in relation to her partner and the context in which she lives (Upadhyay et al. 2014). More recently, other scales have been developed to measure dimensions and related aspects of reproductive autonomy, such as the contraceptive autonomy scale (Senderowicz 2020).

Although women's reproductive autonomy is often considered in relation to their male partner's decision‐making power, it may be questioned whether having more decision‐making power also means that men enjoy reproductive autonomy. For instance, having reproductive autonomy would require them to have access to resources that enable them to make informed decisions, such as comprehensive sexuality education, contraception, skills to communicate about their fertility desires with their partner, and not feeling sociocultural pressure to have children (Ibikunle et al. 2022; Varga 2001; Oni et al. 2024; Gottert et al. 2024). In this way, the same aspects that shape women's reproductive autonomy may also shape men's reproductive autonomy, such as the influence of family members, social networks, sociocultural norms, and governmental policies and regulations on the reproductive autonomy of individuals, couples, and other intimate relationships (Buser et al. 2023; Imhanrenialena et al. 2023; Klu 2023; MacQuarrie and Edmeades 2015). These aspects affecting both men's and women's reproductive autonomy in an intimate relationship can be considered external power dynamics.

Population policies in general, and pronatalist policies in particular, can be shaped by heteronormative and cisnormative norms and values that expect women to both want and be able to become mothers (Graham et al. 2023). Not only can such norms put pressure on women to have children, but they can also increase stigma around infertility and childlessness and neglect the fertility desires of sexual and gender minority populations (Gietel‐Basten 2024; Gerrits et al. 2023). In addition, these norms may assume that all relationships are monogamous, disregarding other intimate partnerships, such as polygamy. These heteronormative and cisnormative norms and values have guided research agendas, and, consequently, certain topics—such as infertility—have been neglected in reproductive autonomy research in sub‐Saharan Africa (Bell et al. 2024).

As fertility research informs population policies, it is important that studies address the reproductive autonomy of all populations, including men, sexual and gender minority populations, and partners in intimate relationships (Strong 2022; Badri, Krumeich, and van den Borne 2023; Stykes 2018; Kabagenyi, Jennings, et al. 2014; Kabagenyi, Ndugga, et al. 2014). Neglecting subpopulations and power dynamics at various interpersonal and societal levels perpetuates reproductive autonomy inequalities and may result in ineffective policies and interventions (Afferri et al. 2022; Graham et al. 2023). For instance, studies focusing solely on women may develop recommendations focused on empowering this subpopulation (e.g., Negash et al. 2023), thereby overlooking the interpersonal and contextual factors also affecting their ability to have reproductive autonomy.

Reproductive justice acknowledges that due to inequalities in society and policies, some individuals and subpopulations have fewer opportunities than other subpopulations to enjoy the right to have or not to have a child under their preferred conditions and to parent in a safe and healthy environment (Ross 2017). As a result, these subpopulations may have less reproductive autonomy. The universality of the reproductive justice framework asserts that every person should have the same capability to enjoy reproductive autonomy (Ross 2017; McGovern et al. 2022). At the same time, scholars have questioned the applicability of individual, rights‐based definitions and measurements of autonomy in general (Chattopadhyay and De Vries 2013), and of reproductive autonomy specifically (Tosam 2020) in communal contexts such as in sub‐Saharan Africa. Alkire (2005) argues that people can also have high reproductive autonomy in communal settings as long as the choices made collectively are in accordance with their own interests, values, and desires. Correspondingly, women have indicated that they feel more empowered when their environment supports their decisions (PSI 2022, in Mandal et al. 2025). However, Khader (2016) notes that “(patriarchal) socialization makes it difficult for women to want or know what would empower them.” Similarly, Jejeebjoy (2000) found that context, especially the role of gender, is one of the most important factors shaping women's autonomy.

The concept of reproductive autonomy is widely used, yet it is often used interchangeably with related concepts, which creates indistinctness about its definition and context‐specific measurements affecting recommendations for policies and interventions. Furthermore, a predominant focus on married women and fertility may have led to a paucity of certain reproductive topics and subpopulations in reproductive autonomy research. The narrow focus of reproductive autonomy on a person's perceived power to decide about and control reproductive matters makes it a useful concept to study the relevant interpersonal and contextual power dynamics, especially in patriarchal societies. However, at the same time, there are mixed indications of its applicability in the, often communal, sub‐Saharan African context. Therefore, to obtain new insights on the applicability of the concept and identify gaps, the objective of this scoping review^1^ is to clarify how the concept of reproductive autonomy has been assessed and applied in fertility research in sub‐Saharan Africa (Munn et al. 2018). This clarification includes the subpopulations studied and the role of gender and other power dynamics internal and external to couples and other intimate relationships.

Reproductive autonomy is context‐specific. For this reason, a regional focus in this scoping review will contribute to context‐specific insights for measurements and recommendations for policies and interventions. In addition to examining communal structures in sub‐Saharan Africa, the literature discusses various sociocultural structures in the region that could be affecting reproductive autonomy, such as marriage type and kinship structures. For instance, women's spousal power in Ghana has been found to differ depending on whether they are in a monogamous or polygynous marriage and, in the latter case, on their seniority within a polygynous marriage (DeRose 2007). Furthermore, women are found to have more decision‐making autonomy, such as the ability to seek health care, in matrilineal kinship structures compared with patrilineal kinship systems in sub‐Saharan Africa (Lowes 2022).

### Review Questions

This review explores the following overall research questions and related subquestions: How is the concept of reproductive autonomy applied and assessed in fertility research in sub‐Saharan Africa?

1. How is reproductive autonomy defined and operationalized in fertility research in sub‐Saharan Africa?
2. Which subpopulations at the individual, couple, and other intimate relationships level are studied in reproductive autonomy research in sub‐Saharan Africa?
3. How are gender and other power dynamics internal and external to couples and other intimate relationships taken into account in reproductive autonomy research in sub‐Saharan Africa?

### Inclusion Criteria

Following the Participants, Concept, and Context framework, this review considered:

- *Participants*: all human populations, meaning people of all genders, sexual orientations, and ages, at the individual, couple, and other intimate relationships level;
- *Concept*: studies that assess and apply reproductive autonomy in fertility research;
- *Context*: studies that focus on sub‐Saharan Africa and its countries and subregions as classified by the United Nations and the MeSH descriptor for Africa South of the Sahara (UN DESA Statistics Division 2024; National Library of Medicine 2024). This includes multicountry studies in which one or multiple countries or subregions in sub‐Saharan Africa are analyzed separately.

This scoping review considered empirical study designs for inclusion, including quantitative, qualitative, and mixed methods. Studies had to be peer‐reviewed, academic articles published since 1994. The year 1994 was chosen because the importance of reproductive autonomy was globally acknowledged in the ICPD Programme of Action in 1994. Due to the language limitations of the authors, we intended to initially include non‐English studies if they had an English title and abstract but to exclude them at the stage of full‐text screening. However, only English studies were selected at the title and abstract stage.

## METHODS

The scoping review was conducted in accordance with scoping review guidance, namely, the Joanna Briggs Institute (JBI) guidelines (Peters et al. 2024), the process principles proposed by Arksey and O'Malley (2005) and further developed by Levac, Colquhoun, and O'Brien (2010), and the Preferred Reporting Items for Systematic Reviews and Meta‐Analysis (PRISMA) extension for scoping reviews (PRIMA‐ScR) checklist (Tricco et al. 2018). More details can be found in the protocol, which has been registered with the Open Science Framework (OSF) under registration number osf.io/7243r.

**FIGURE 1:**
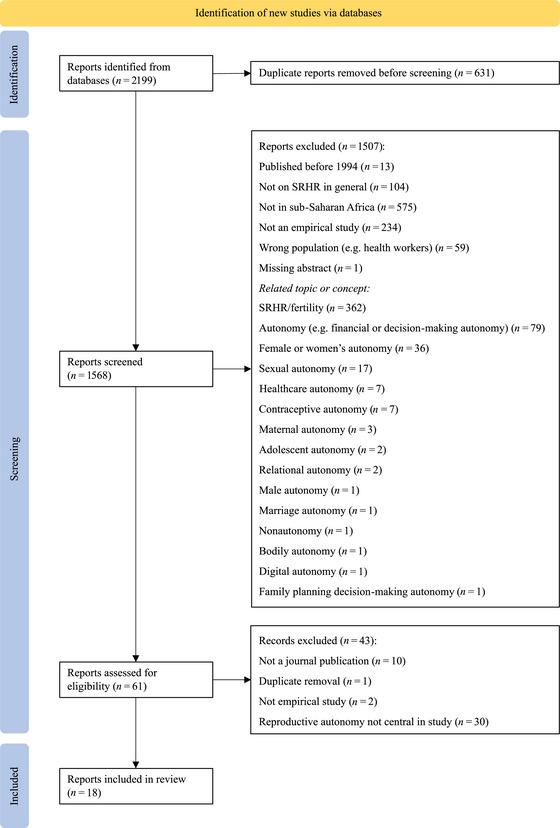
Prisma flow diagram of included studies SOURCE: Haddaway et al. (2022)

The search strategy aimed to locate peer‐reviewed published empirical studies. As all populations are included, and we were only interested in one concept, that is, “reproductive autonomy,” the development of the search strategy was mostly concerned with the inclusion of all relevant geographical locations (see online Appendix: Supplemental Materials A1). We selected nine databases together covering a variety of disciplines in which fertility research is conducted, including demography, sociology, public health, and psychology: Academic Search Premier (EBSCOhost), APA PsychInfo (EBSCOhost), CINAHL (EBSCOhost), EMBASE, IBSS (ProQuest), PubMed (NIH), Scopus, SOCIndex (EBSCOhost), and Web of Science (Clarivate). Additionally, we searched through four African journal databases and we manually searched for articles in the *Pan African Medical Journal* using the keyword “reproductive.” As shown in Figure 1, we identified 2199 articles, which we uploaded to the bibliographic citation management software EndNote version 21. We removed 631 duplicates. Then, we imported the 1568 citation details into Rayyan, which is a free web application to facilitate the screening process for researchers working together on a scoping review (Ouzzani et al. 2016).

In the first round, we screened the titles and abstracts of the 1568 articles. In total, 61 articles were included for full‐text review and 1507 articles were excluded. Figure 1 shows the reasons for exclusion. Articles received one label for exclusion in order of appearance, even when multiple reasons were applied, such as “not empirical study” and “wrong population.” Studies that did not focus specifically on countries in sub‐Saharan Africa—such as those referring more broadly to “developing countries”—were excluded. “Not empirical studies” included, among other things, systematic literature reviews and human rights perspectives on reproductive autonomy, often focusing on ethics, marginalized populations, and contextual aspects relevant to a better understanding of reproductive justice. Studies were also excluded when they addressed perceptions about other populations, for instance, health workers’ perceptions of their clients’ reproductive autonomy. Furthermore, we excluded articles that did not explicitly apply the concept of “reproductive autonomy,” even if their themes aligned with the concept of reproductive autonomy as discussed in the introduction. The label “SRHR (sexual and reproductive health and rights)/fertility” was given to studies that focused on fertility and SRHR outcomes, including studies addressing reproductive empowerment, coercion, agency, power, control, choice, and decision‐making. Furthermore, studies were excluded if they focused on subconcepts such as “contraceptive autonomy,” on more general concepts such as “autonomy,” or on related concepts such as financial autonomy and how these may affect fertility or SRHR outcomes.

In the next step, we retrieved the full texts of the 61 included articles using EndNote and online searching, and we imported the full‐text pdf documents into ATLAS.ti Web (https://web.atlasti.com/). Two authors were directly contacted and sent us their publications because we could not access them via our university accounts. In ATLAS.ti Web, we manually created codes for each research subquestion to streamline the full‐text review.

In the first round of the full‐text review, the first two authors excluded 37 articles, included 10, and were conflicted about 14. After discussion, we concluded that 18 texts met the inclusion criteria and excluded 43 texts. Figure 1 shows the reasons for exclusion in the round of full‐text review. Texts were excluded because they were duplicates or not an empirical study or an academic peer‐reviewed journal article. In addition, we excluded texts if they did not define, operationalize, or assess reproductive autonomy as a concept in‐depth. For instance, some articles referred to reproductive autonomy only as part of the problem statement in their background or as an implication in their discussion or conclusion.

Next, the two reviewers individually extracted the data using the data extraction tool (see online Appendix: Supplemental Materials A2) and compared their findings. Subsequently, we described the characteristics and key findings of each article in a table and compared them for similarities, variations, and gaps.

## RESULTS

### Overview of Publications

Table 1 shows the aim, study population, country focus, sample size, and methodology of the 18 included publications. The majority used quantitative methods (*n* = 15), and only a few used qualitative (*n* = 2) or mixed methods (*n* = 1). Most are recent: three were published between 1994 and 2016, 15 since 2017, and nine since 2021.

**TABLE 1:** Overview of included studies

	Authors	Aims of the study	Country	Population and sample size	Methodology
1	Bell et al. (2024)	(1) Determine the full distribution of desired pregnancy timing among recent births and current pregnancies and evaluate sociodemographic and reproductive factors associated with having a later‐than‐desired pregnancy; (2) examine how these factors differ when comparing those who experienced a pregnancy later than desired to those who experienced their pregnancy when they desired	Uganda	A nationally representative sample of reproductive‐aged women who were currently pregnant or who had ever given birth in Uganda (*n* = 3311)	Quantitative (survey)
2	Boyce et al. (2023)	Assess the reliability and validity of a new measure of the social acceptability of IPV when it is used to exert control over a wife's agency, sexuality, and reproductive autonomy (IPV‐ASRA Social Norms scale)	Niger	Married adolescent girls (aged 16–22) and their husbands (aged 18–66) in rural Niger (*n* = 559 husband–wife dyads)	Quantitative (RCT survey)
3	Decker et al. (2021)	(1) Describe a set of gendered relationship power imbalances and their interplay; (2) examine their associations with modern contraceptive use and proximal threats to reproductive autonomy	Côte d'Ivoire, Kenya, Nigeria	Unmarried, currently partnered adolescent girls, and young adult women aged 15–24 in Abidjan, Côte d'Ivoire (*n* = 555 in 2018–2019), Nairobi, Kenya (*n* = 332 in 2019), and Lagos, Nigeria (*n* = 179 in 2020)	Quantitative (survey)
4	DeRose and Ezeh (2005)	(1) Test the relative weight of husbands’ and wives’ characteristics on the fertility intention of both partners; (2) test whether the influence of husbands’ characteristics on wives’ fertility intentions decreased, stayed constant, or increased during the first decade of rapid fertility decline; (3) explore whether patterns of relative spousal influence observed in the country also occur in urban areas where fertility decline has progressed further	Ghana	A nationally representative sample of women aged 15–49 years and their co‐resident spouses, who were men aged 15–59 (couple records: n = 1010 in 1988; *n* = 547 in 1993; *n* = 629 in 1998)	Quantitative (DHS survey)
5	Nii‐Amoo Dodoo, Horne, and Biney (2014)	Provide some insight into the efficacy of female education for mitigating the effect of bridewealth on normative constraints on a woman's reproductive (and thereby sexual) autonomy	Ghana	Women aged 18 years and over living in patrilineal communities in Ghana (*n* = 276; six conditions, 46 per condition)	Quantitative (vignette study)
6	Dodoo et al. (2019)	(1) Examine the association between ethnicity and sexual and reproductive autonomy in a primarily indigenous Ga community in Accra, Ghana; (2) investigate the link between women's financial autonomy and their sexual and reproductive autonomy in this context	Ghana	Women aged 15–49 years who were married or in a consensual union living in the Accra region (urban, poor context), Ghana (*n* = 172)	Quantitative (survey)
7	Dodoo, Horne, and Dodoo (2020)	Assess the implications of a central component of African marriage, bridewealth, for norms constraining wives’ reproductive autonomy	Ghana	Men (*n* = 1152) and women (*n* = 1248) aged 18 or older in matrilineal and patrilineal communities in the Eastern region of Ghana (total *n* = 2400; 83 participants in polygamous relationships)	Quantitative (vignette study)
8	Horne, Nii‐Amoo Dodoo, and Naa (2013)	Assess a theoretical prediction regarding the causal link between bridewealth and normative constraints on women's reproductive autonomy	Ghana	Women aged 18 years and over living in a rural area (the North Tongu district in the Volta region) of Ghana (*n* = 276; six conditions, 46 per condition)	Quantitative (vignette study)
9	Kunesh et al. (2024)	Further understand the relationship between age‐disparate relationships at first sex and reproductive autonomy, reproductive empowerment, contraception coercion, and sexual violence among Rwandan adolescent girls and young women	Rwanda	In‐school youth ages 12–19 at enrollment with a focus on those who reported sexual activity in Rwanda (*n* = 1319)	Quantitative (RCT survey)
10	Loll et al. (2019)	(1) Examine whether reproductive autonomy within a partnership is associated with modern contraceptive use at last sex among young women in Ghana; (2) examine the associations between social context variables and contraceptive use to explore their influence on the relationship between reproductive autonomy and modern contraceptive use at last sex	Ghana	Women aged 15–24 who are currently in a relationship and who were not pregnant or intended to get pregnant, living in Kumasi and Accra (urban areas in Ghana) (*n* = 325)	Quantitative (survey)
11	Loll et al. (2020)	(1) Examine who had the most say in the outcome of young Ghanaian women's last pregnancy and whether this correlated with their level of reproductive autonomy; (2) investigate the role of social context through inclusion of the variables of social approval for adolescent sexual and reproductive health (SRH) and stigma toward adolescent SRH	Ghana	Previously pregnant women aged 15–24 years who are currently in a relationship, living in Kumasi and Accra (urban areas in Ghana) (*n* = 380)	Quantitative (survey)
12	Loll et al. (2021)	Understand the sociodemographic, reproductive experience, and social factors associated with reproductive autonomy among young Ghanaian women	Ghana	Women aged 15–24 who are currently in a romantic or sexual relationship, living in Kumasi and Accra (urban areas in Ghana) (*n* = 516)	Quantitative (survey)
13	Obare et al. (2022)	Explore how husbands’ experiences with a self‐use product developed for women might influence women's rights and autonomy regarding their reproductive decisions and behavior in an environment characterized by gender imbalances around such decisions	Kenya, Nigeria, Senegal	Women aged 18–35 years (*n* = 174: 60 Kenya, 58 Nigeria, 56 Senegal) and their husbands (*n* = 10: three Kenya, three Nigeria, four Senegal)	Mixed methods (quantitative, structured interviews, and in‐depth interviews)
14	Osuafor and Okoli (2021)	Identify socioeconomic factors that influence unionized women's decision‐making on sex and family size, using the theory of gender and power	South Africa	Heterosexual married and cohabiting women aged 18–49 years in Mahikeng, South Africa (*n* = 568)	Quantitative (survey)
15	Princewill, De Clercq, et al. (2017)	Explore the impact of education on married Ikwerre women's reproductive autonomy	Nigeria	Married Ikwerre women aged 22–60 years in monogamous and polygynous marriages in Rivers State, Nigeria (*n* = 34)	Qualitative (semistructured in‐depth interviews)
16	Princewill, Jegede, et al. (2017)	(1) Examine how married Ikwerre women understand reproductive rights and autonomy; (2) examine what affects the exercise of their reproductive rights in their marital homes	Nigeria	Married Ikwerre women aged 22–60 years in monogamous and polygynous marriages in Rivers State, Nigeria (*n* = 34 interviews and six focus group discussions, each with four to five women)	Qualitative (semistructured in‐depth interviews and focus group discussions)
17	Svallfors et al. (2024)	Investigate the links between armed conflict, perceptions of neighborhood insecurity, and attitudes toward women's and girls’ reproductive autonomy in Nigeria	Nigeria	Men (*n* = 559) and women (n = 534) aged 18 years and above (total *n* = 1093)	Quantitative (survey and UGDP‐GED dataset)
18	Wollum et al. (2023)	(1) Determine the reliability and construct validity of the Reproductive Autonomy Scale and two of its subscales in a population of partnered women (aged 16–47 years) in a rural community in Central Malawi; (2) determine whether reproductive autonomy was associated with contraceptive use among women in partnerships	Malawi	Women who had ever had sex aged 16–47 in (rural) Central Malawi, excluding those who were single, currently pregnant, had reached menopause or were sterilized (*n* = 695)	Quantitative (survey)

**FIGURE 2:**
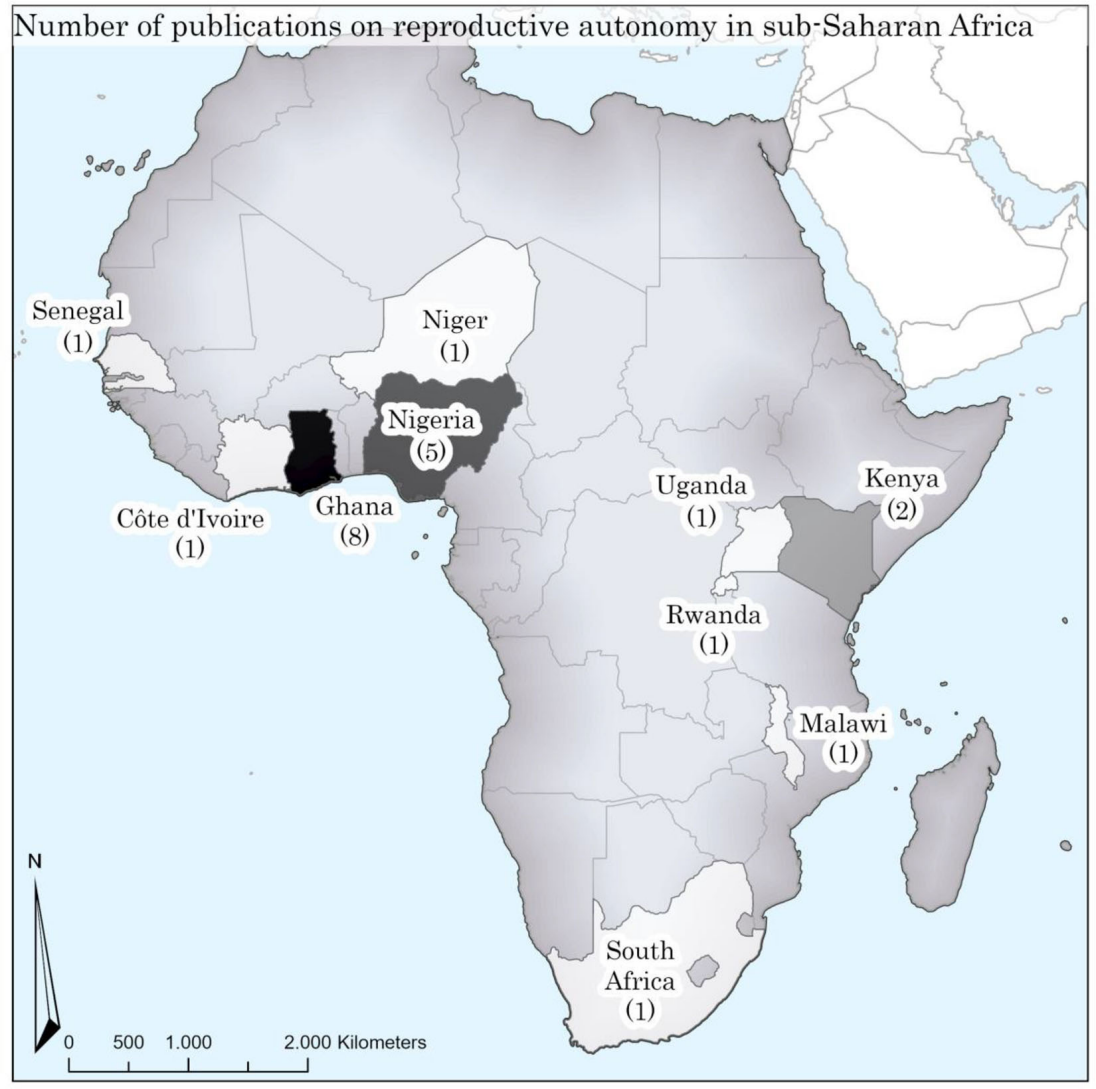
Selected number of publications on reproductive autonomy in sub‐Saharan Africa

The publications are geographically spread over 10 countries (see map in Figure 2). Two studies (Decker et al. 2021; Obare et al. 2022) covered three countries. The articles report on Western Africa (*n* = 16), including Ghana (*n* = 8) and Nigeria (*n* = 5), Eastern Africa (*n* = 4), and Southern Africa (*n* = 2). The high number in Ghana is mostly explained by three publications by Dana Loll and co‐authors and four publications by F. Nii Amoo Dodoo, Naa D. Dodoo, and Christine Horne.

### Definition of Reproductive Autonomy

Table 2 shows the key findings in answer to the research questions. Some publications provided a clear definition (*n* = 10) or a description (*n* = 4) of reproductive autonomy, while others did not define the concept (*n* = 4).

**TABLE 2:** Overview of key findings

	Authors	Definition of reproductive autonomy	Operationalization of reproductive autonomy	Subpopulations	Gender and other power dynamics
1	Bell et al. (2024)	“The ability to achieve one's desired fertility intentions, including the number and timing of children”	Outcome: “Did you want to become pregnant earlier”? Yes/No “How many months did it take for you to become pregnant?”	*Individual level*: Women aged 15–49	No, the focus is on the individual characteristics
2	Boyce, et al. (2023)	No—but seems to position reproductive autonomy as the opposite of reproductive coercion/control	Scale item: “People in your community believe that… a husband is justified in hitting or beating his wife if she uses a family planning method without informing him” Disagree, somewhat agree, agree	*Individual level*: Husbands of adolescent wives	*External power dynamics*: Assessment of social norms regarding intimate partner violence. Scale items about challenging husband authority were related to husband's perpetration of intimate partner violence against his wife
3	Decker et al. (2021)	No definition provided	Reproductive characteristics/threats to reproductive autonomy: (1) stealthing/condom removal; (2) reproductive coercion; (3) high capability of avoiding unwanted sex; (4) confidence in contraception; (5) fear‐related contraceptive procurement	*Individual level*: Unmarried, currently partnered women aged 15–24	*Internal power dynamics*: Relationship power disparities. Power imbalances were linked with coercive sexual/reproductive health experiences
4	DeRose and Ezeh (2005)	“The ability to actualize fertility intentions”	The effects of partner's influence on own desire and intention. Dependent variable “fertility intention”: “Would you like to have a (another) child or would you prefer not to have any (more) children?” Yes/No	*Couple and intimate partner level*: Women, including those in polygynous relationships, and their co‐resident spouses	*Internal power dynamics*: Relative spousal power: men's education determines both men's and women's fertility intentions to a greater extent than women's education does
5	Nii‐Amoo Dodoo, Horne, and Biney (2014)	No definition—it does indicate that community members can constrain people's reproductive behaviors	*Vignette, six experimental conditions*: No/partial/complete bridewealth related to business or reproduction, that is, using contraception without telling husband. Behavior of the women (10 “very right” to 1 “very wrong”)	*Individual level*: Women aged 18 years and above	*External power dynamics* influencing internal power dynamics: Bride payment (as a culturally forced obligation) directly reduces a woman's autonomy because it transfers authority over the number and timing of her children to her husband and indirectly via social norms
6	Dodoo et al. (2019)	Not clearly defined— reproductive autonomy implies “freedom from reproductive coercion, interference with reproductive decision‐making and behavior” and assesses “self‐efficacy regarding contraception, pregnancy, and childbearing”	*Dependent variables*: “Perceived reproductive autonomy”: whether women think their partners would (1) get angry or (2) think they were having sex with other people if they asked him to use a condom “Actual reproductive autonomy”: how comfortable women felt voicing their opinions despite opposing views from their partners in relation to (1) whether to use contraception, (2) which type of contraception to use, and (3) the number of children to have	*Individual level*: Women aged 15–49 years who were married or in a consensual union, including polygamous unions	*Internal power dynamics*: Perceptions of women about voicing their opinions and response partner. Financial autonomy does not increase sexual autonomy, perceived reproductive autonomy, and actual reproductive autonomy. Marital power, agreement with partners about reproductive issues, and marital duration were more associated with sexual and reproductive autonomy
7	Dodoo, Horne, and Dodoo (2020)	No definition provided	Vignette: “[…] One day the man found out that the woman had been [using contraception] without telling him.” Normative expectations and attitudes across conditions: (1) bridewealth: no payment, some payment, full payment; (2) normative expectations from husband, husband's family, men, wife's family, women participants	*Individual level*: Men and women aged 18 or above	*External power dynamics*: Perceived norms of various community members on the role of bridewealth on reproductive autonomy woman Finding that women experience normative constraints on their reproductive decision‐making
8	Horne, Nii‐Amoo Dodoo, and Naa (2013)	Not clearly defined—it does indicate that community members can constrain people's reproductive behaviors and “*constricted reproductive autonomy*” as a “lack of control over own reproductive behavior”	Vignette: “[…] One day the man found out that the woman had been [using contraception] without telling him.” (how right or wrong the woman's behavior was) NEXT PART “When the man found out that the woman had been [using contraception] he was very angry. When he came home he beat her.” (how right or wrong the man was for beating the woman)	*Individual level*: Women aged 18 or above	*External power dynamics*: Bridewealth—and its effects on norms constraining women's reproductive autonomy. Finding that the husband enforces norms, this is an example of how internal power dynamics are a result of external power dynamics
9	Kunesh et al. (2024)	*Decision‐making reproductive autonomy*: “the power to make reproductive decisions without influence from partners and/or family members”	The primary outcome, reproductive autonomy, was adapted from the reproductive autonomy‐decision‐making subscale of the Reproductive Autonomy Scale developed by Upadhyay et al. (2014)	*Individual level*: In‐school youth ages 12–19	*Internal power dynamics*: Adolescent girls and young women are significantly less likely to provide consent at first sex when engaging with an age‐disparate partner. There was no association found between age‐disparate relationships at first sex and the participant's power to make decisions regarding contraception, pregnancy, and childbearing
10	Loll et al. (2019)	“Having the power to decide about and control matters associated with contraceptive use, pregnancy, and childbearing”	Reproductive autonomy within a partnership—a modified version of the Reproductive Autonomy Scale developed by Upadhyay et al. (2014) using two of the three subscales: (1) reproductive autonomy decision‐making: you, not your partner, (1) have the most say about whether you would use a method to prevent pregnancy; (2) have the most say about when you have a baby in your life; (3) would have the most say about whether you would raise the child, seek adoptive parents, or have an abortion if you became pregnant and it was unplanned;
			(2) reproductive autonomy communication: 1, My partner would support me if I wanted to use a method to prevent pregnancy; 2, If I didn't want to have sex, I could tell my partner; 3, If I really did not want to become pregnant, I could get my partner to agree with me	*Individual level*: Women aged 15–24 who are currently in a relationship	*Internal power dynamics*: Perceptions of young women regarding their partner: This decision‐making subscale does not indicate whether the partner was actually informed and involved in the decision‐making process. *External power dynamics*: Social context may affect associations between reproductive autonomy, decision‐making, and modern contraceptive use at last sex
11	Loll et al. (2020)	“Having the power to decide about and control matters associated with contraceptive use, pregnancy, and childbearing,” and “the extent to which [she] is able to execute [her] reproductive decisions free from undue influence from her partner, family, community, and government”	Same as Loll et al. (2019)	*Individual level*: Previously pregnant women aged 15–24 years who are currently in a relationship	*Internal power dynamics*: Perceptions of young women regarding their partner. Decision‐making reproductive autonomy, but not communication reproductive autonomy, was associated with who was involved in pregnancy decision‐making during the most recent pregnancy *External power dynamics*: Young women who reported more of community members, family, and friends as being supportive were more likely to have had someone else make the pregnancy decision
12	Loll et al. (2021)	“Having the power to decide about and control matters associated with contraceptive use, pregnancy, and childbearing” and “ability to choose […] without undue influence from men, healthcare providers, the government, the international development community, or religious doctrine”	Same as Loll et al. (2019)	*Individual level*: Women aged 15–24 who are currently in a relationship	*Internal power dynamics*: On average, young women were more comfortable discussing their reproductive decisions with their partners (communication reproductive autonomy) than having the most say in the decision (decision‐making reproductive autonomy) *External power dynamics*: Individual level variables reflect both individual and social context characteristics Importance of education, religion, and reproductive history covariates to reproductive autonomy
13	Obare et al. (2022)	No definition provided	Women's autonomy in the context of decision‐making regarding contraception and choice of the method, women's ability to self‐use the method and manage associated challenges, and husbands’ role in discontinuation of the method and contraceptive switching	*Couple level*: Lactating mothers seeking family planning services and their husbands	*Internal power dynamics*: How husbands’ experiences influence women's reproductive autonomy acknowledging the context of gender imbalances around such decisions
14	Osuafor and Okoli (2021)	No definition provided	*Dependent variables*: Decision‐making on (1) sex and (2) number of children (“Can a woman decide on the number of children she wants to give birth to?” Yes or no)	*Individual level*: Heterosexual married and cohabiting women aged 18–49 years	*Internal power dynamics*: Type of marriage influences women's autonomy on sex and family size (civil vs. religious and traditional) *External power dynamics*: Attributed to strong gender equality initiatives in South Africa; traditional patriarchal values on sexual and reproductive decision‐making create an inability for women to decide on family size
15	Princewill, De Clercq, et al. (2017)	“A woman's ability or freedom to exercise her reproductive rights” (article uses reproductive autonomy and reproductive rights interchangeably)	Semistructured research guide on the role of education in the exercise of women's reproductive autonomy. Derived subthemes: (1) reproductive autonomy and family wellbeing; (2) culture of absolute respect for men; (3) financial independence; (4) change in social status	*Individual level*: Married women aged 22–60 years in monogamous and polygynous marriages	*Internal power dynamics*: Husbands’ education is important for women's reproductive autonomy because men act as gatekeepers of cultural norms and practices (subsumed under perceived marital obligations); The culture of absolute respect for men was a major hindrance to women's reproductive autonomy. Educated women were able to use their reproductive autonomy (and having financial means to achieve this) in a subtle way without annoying their husband
16	Princewill, Jegede, et al. (2017)	“The ability or freedom of a woman to exercise her reproductive rights”	*Topics in the interview guide*: Personal understanding of reproductive rights, ability to exercise these reproductive rights in the family, understanding of autonomy, decision‐making regarding sexual relationships, and the right to refuse sex as well as its consequences	*Individual level*: Married women aged 22–60 years in monogamous and polygynous marriages	*Internal power dynamics*: Marriage directly obligates women to have sex with their husbands and bear children (as many as the husband wishes). Education has helped educated women to secretly go for family planning without their husband's consent because they work and have their own money *External power dynamics*: The role of the mother‐in‐law and older women in the compound reinforces the submissiveness of women to their husbands
17	Svallfors et al. (2024)	“Their capacity to make free and informed decisions about whether, when, and with whom to partner and have children”	*Dependent variables*: Five indicators to broadly reflect attitudes to reproductive autonomy in terms of women's and girls’ decision‐making related to childbearing and relationships: (1) contraception; (2) abortion; (3) marital decision‐making; (4) delayed childbearing; (5) early marriage for security	*Individual level*: Men's and women's perceptions about women's and girls’ reproductive autonomy	*External power dynamics*: Findings suggest that conflict and insecurity pose a threat to, but also facilitate opportunities for women's reproductive autonomy
18	Wollum et al. (2023)	“The power to decide about and control matters associated with contraceptive use, pregnancy, and childbearing”	*Two subscales of the Reproductive Autonomy Scale* (Upadhyay et al, 2014): (1) freedom from coercion (5 items); (2) communication (5 items)	*Individual level*: Women who had ever had sex aged 16–47	*Internal power dynamics*: Higher levels of freedom from coercion and in some cases, higher levels of communication, were associated with contraceptive use

Five articles used the definition by Upadhyay et al. (2014), which reasons from an individualistic perspective: “the power to decide about and control matters associated with contraceptive use, pregnancy, and childbearing.” However, three of these publications added a component that more explicitly highlights the role of interpersonal and contextual power dynamics by indicating that reproductive autonomy concerns having the ability to make decisions without influence from partners, family, healthcare providers, the community, government, the international development community, and religious doctrine (Kunesh et al. 2024; Loll et al. 2020, 2021). Of the other five definitions, two focused more narrowly on fertility intentions only (Bell et al. 2024; Derose and Ezeh 2005), two referred to Purdy (2006) by emphasizing that reproductive autonomy concerns a woman's ability to exercise her reproductive rights (Princewill, De Clercq, et al. 2017; Princewill, Jegede, et al. 2017), and one included a broader definition focusing not only on making decisions about children but also on having a partner (Svallfors et al. 2024).

The four publications that provided a description positioned reproductive autonomy as negative freedom—being free from reproductive coercion, interference, or control in general (Boyce et al. 2023; Dodoo et al. 2019), or from restrictions imposed by community members in particular (Nii‐Amoo Dodoo, Horne, and Biney 2014; Horne, Nii‐Amoo Dodoo, and Naa 2013).

Although some definitions focused on individuals or women only, none explicitly focused on partners in intimate relationships. Furthermore, the definitions did not explicitly distinguish between power dynamics with a partner, those in other immediate and distant interpersonal relationships, and those arising from contextual aspects.

### Operationalization of Reproductive Autonomy

In line with the provided definitions, reproductive autonomy was operationalized by addressing the following components: pregnancy and fertility (*n* = 14), contraception (*n* = 13), and unwanted sex (*n* = 6). Depending on its definition, childbearing could be considered part of pregnancy and fertility, that is, when to have a baby in your life, and being able to decide to raise a child, seek adoptive parents, or have an abortion (Upadhyay et al. 2014). However, other imaginable operationalizations, such as being able to decide to give birth under the circumstances of one's choosing, were not included in the publications. The two qualitative studies did not specify which components of reproductive autonomy were discussed during their interviews (Princewill, De Clercq, et al. 2017; Princewill, Jegede, et al. 2017).

Five articles applied one or more subscales of the reproductive autonomy scale by Upadhyay et al. (2014). Four of them used the reproductive autonomy “decision‐making subscale,” which measures perceptions on who decides about contraceptive use, pregnancy, and childbearing (Upadhyay et al. 2014). As pointed out by Loll et al. (2019), this subscale does not indicate whether the partner was actually informed and involved in the decision‐making process. The relevance of operationalizing actual involvement is supported by the qualitative study of Princewill, De Clercq, et al. (2017) who found that women may state that their partner is supposed to be the decision‐maker, yet still value their own education and financial resources enabling them to use covert contraception. However, this is a different interpretation of “actual reproductive autonomy” compared with Dodoo et al. (2019), who measured this as how comfortable women felt communicating with their partners about contraception and fertility. Upadhyay et al. (2014) considered this in their “communication subscale.”

Most publications measured reproductive autonomy in relation to power dynamics, including the partner or other interpersonal relationships (*n* = 11), social norms around intimate partner violence (IPV), marital obligations, and bridewealth (*n* = 4), and contextual aspects such as conflicts and insecurity, and the rise in civil marriages (*n* = 2).

### Inclusion of Subpopulations at the Individual and Intimate Relationship Level

The publications included both individuals and couples as participants from different communities and study settings. At the individual level, study participants were often women aged 15–49 who were married—including polygynous unions—or otherwise partnered or sexually active, which enabled the study of interpersonal power dynamics and reproductive outcomes. Relatively few publications included men and couples or other intimate relationships as a unit of analysis. Two articles researched the reproductive autonomy and fertility intentions of both men and women (Derose and Ezeh 2005; Kunesh et al. 2024). In other examples, men were included in their role as a community member (Svallfors et al. 2024; Dodoo, Horne, and Dodoo 2020) or as a husband in relation to women's reproductive autonomy (Obare et al. 2022; Boyce et al. 2023). At the couple level, only two of the 18 publications focused on both women and their intimate partners (Obare et al. 2022; Derose and Ezeh 2005). As indicated by Obare et al. (2022), power imbalances—and the related fear of IPV or other negative consequences for the female partner—prevent research from focusing on both partners or even multiple partners within a polygynous marriage. Indirectly, Boyce et al. (2023) were able to study couples by linking husband's responses to women's reports of IPV.

Osuafor and Okoli (2021) specifically mentioned that their participants were heterosexual; implicitly all publications appeared to focus on heterosexual relationships. No study expressly mentioned having included sexual and gender minority populations. Likewise, most publications did not explicitly study other vulnerable or marginalized populations, such as people with disabilities. Exceptions were one study that focused on in‐school youth aged 12–19 (Kunesh et al. 2024) and one on reproductive autonomy in conflict situations (Svallfors et al. 2024). Accordingly, the publications hardly addressed laws or policies that could unequally affect the reproductive autonomy of certain populations.

### Inclusion of Gender and Other Power Dynamics

This section provides a synopsis of the articles in relation to the role of gender and other power dynamics in understanding reproductive autonomy. The findings show that in the sub‐Saharan African context, the communal society could be considered the foundation, with its legal and sociocultural context often rooted in patriarchy and pronatalism. These are reflected in traditions, norms, and values at the community level, such as payment of bridewealth in traditional or cultural marriage, including polygynous marriages. Patriarchal values may have a stronger hold in traditional marriages compared with civil marriages, which may show more gender equality (Osuafor and Okoli 2021). The societal context can also involve conflicts and insecurities, creating both opportunities and threats to reproductive autonomy (Svallfors et al. 2024).

Whenever a couple marries, especially in the case of traditional marriage and payment of bridewealth, the woman's reproductive autonomy reduces as she transfers her fertility authority to her husband (Nii‐Amoo Dodoo, Horne, and Biney 2014). Interpersonal relationships, such as with a mother‐in‐law, and community members at large, reinforce traditions by ensuring that the married couple abides by their marital obligations (Dodoo, Horne, and Dodoo 2020; Princewill, Jegede, et al. 2017).

A woman may show low reproductive autonomy, as explained by various sociodemographic and reproductive factors (Bell et al. 2024; Loll et al. 2021). Findings show that financial autonomy may not necessarily result in higher reproductive autonomy for women (Dodoo et al. 2019) and that husbands’ experiences of contraception influence women's reproductive autonomy (Obare et al. 2022). Similarly, education for men seems more influential for women's reproductive autonomy and fertility intentions than women's own educational level (Derose and Ezeh 2005; Princewill, De Clercq, et al. 2017), although education may support women financially and enable them to use covert contraception (Princewill, Jegede, et al. 2017). Furthermore, in urban areas, education may be more important for explaining fertility decline and women may have more autonomy to decide their family size than in rural areas (DeRose and Ezeh 2005; Osuafor and Okoli 2021). Marital duration was also found to be positively associated with reproductive autonomy (Dodoo et al. 2019). The influence of men on their female partner's reproductive autonomy is explained by men acting as gatekeepers of cultural norms and practices (Princewill, De Clercq, et al. 2017) and by relational power disparities, such as age disparity, which capacitate men to have control and use coercion (Kunesh et al. 2024; Decker et al. 2021). A woman may challenge her husband's authority, for instance, by using contraception against her husband's will. However, in such a case, the husband may use IPV to reinforce social norms and, thus, restore the power balance in their relationship. When a bridewealth has been paid, women themselves can feel IPV is justified, and men can feel that people in their community would justify the use of IPV too (Boyce et al. 2023; Horne, Nii‐Amoo Dodoo, and Naa 2013). Princewill, Jegede, et al. (2017) indicate that women also fear that challenging a husband's authority may lead them to end up in polygynous marriages or divorce.

The resulting reproductive autonomy enjoyed by the partners in the intimate relationship, shaped by interpersonal and contextual power dynamics, may result in various reproductive outcomes, including contraceptive use and pregnancy (Loll et al. 2019, 2020; Wollum et al. 2023).

**FIGURE 3:**
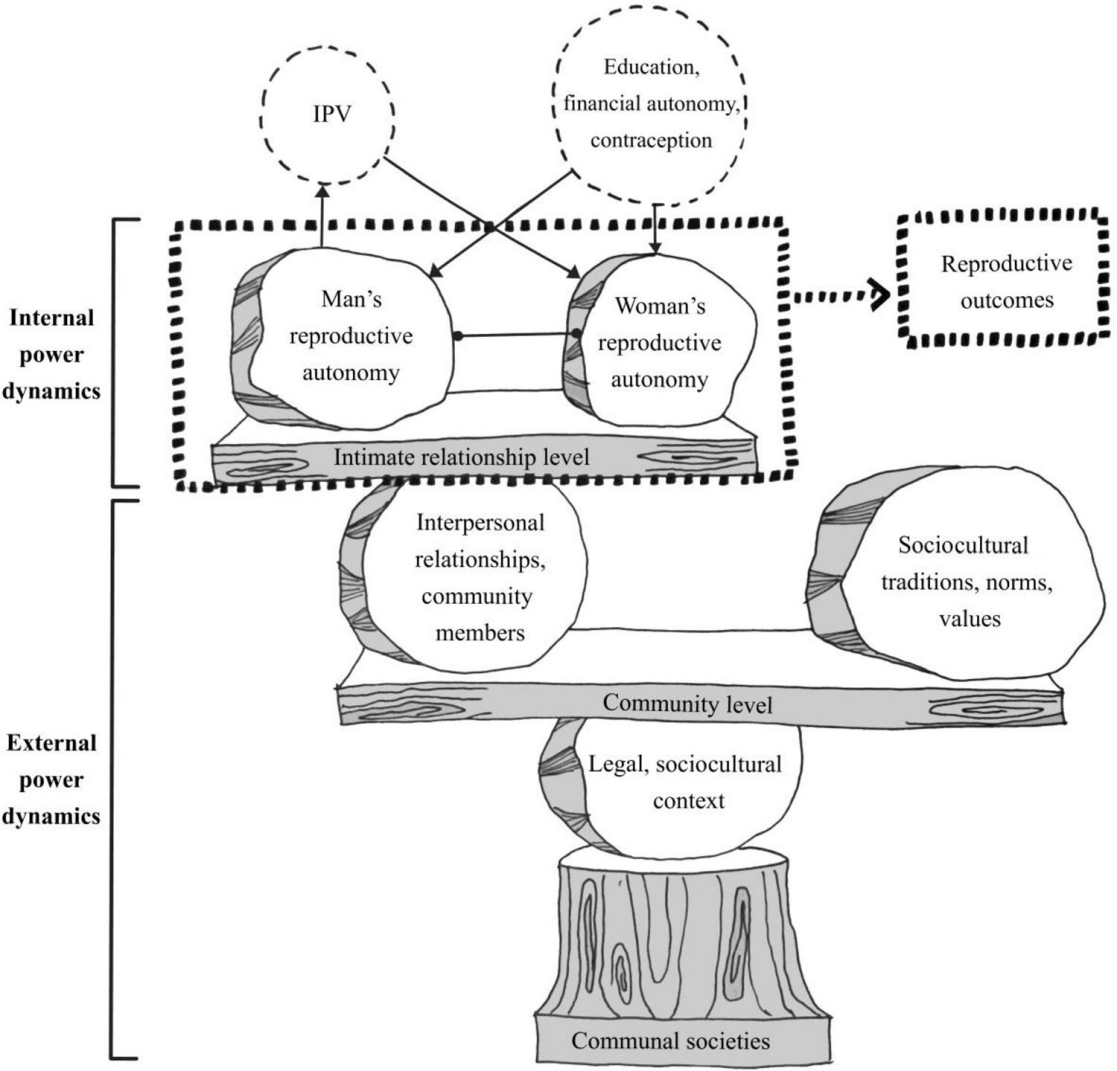
Visual summary of selected texts metaphorically representing a “balance” SOURCE: Inspired by a photo of stones kept in balance on wooden planks and a tree trunk on the beach by Dimitri Otis / Getty Images.

Figure 3 visually represents a summary of these findings using a “balance” as a metaphor for the gender and other power dynamics involved. The balance shows how the community level reinforces power structures within intimate relationships to maintain current social power structures at the societal level. In this way, the reproductive autonomy of partners in intimate relationships in general, and of certain people in particular—women in the case of the included publications—is controlled to sustain this balance. In accordance with the foci of the included publications, the visual summary reflects a heteronormative situation of a man and a woman in an intimate partner relationship, thereby, acknowledging that intimate relationships can encompass various sexual orientations and number of partners. Even though most publications focused on women's reproductive autonomy and their reproductive outcomes, the visual assumes that these outcomes are not solely the result of women's reproductive autonomy but rather a result of the reproductive autonomy enjoyed by the partners in an intimate relationship.

## DISCUSSION

This scoping review aimed to clarify how the concept of reproductive autonomy has been assessed and applied in fertility research in sub‐Saharan Africa. On the basis of the 18 included publications, it can be concluded that most empirical studies were quantitative and that they primarily focused on the reproductive autonomy of women aged 15–49 in an intimate partnership. Overall, the definitions typically adopt an individualistic perspective, rarely explicating the influence of partner dynamics, other interpersonal relations, and context. However, reproductive autonomy is often operationalized in relation to such power dynamics. These power dynamics demonstrate how, in order to preserve existing social power structures at the societal level, the community level reinforces power structures within close connections. Therefore, to maintain this equilibrium, the reproductive autonomy of individuals in intimate relationships is restricted in general, but particularly for women, as highlighted by the included studies.

The review found reproductive autonomy to encompass many aspects that are often conceptualized differently or not at all. As a result, many articles studying components or related issues that are relevant for understanding reproductive autonomy in sub‐Saharan Africa were excluded from the selection process. Similarly, by only including empirical publications, we excluded human rights articles, which could have informed us about policies and laws that shape the reproductive autonomy of vulnerable and marginalized populations in sub‐Saharan Africa. Conversely, focusing only on reproductive autonomy as a concept in empirical work has provided various insights and identified gaps in the literature.

A first insight from this scoping review is that most of the included publications are recent and that their number per year has been increasing. This may indicate that more publications can be expected in years to come. The definition and measurement of reproductive autonomy as provided by Upadhyay et al. (2014) have been influential for, and seem to have prompted, the number of publications on reproductive autonomy in sub‐Saharan Africa. We noticed that their definition and measurement take an individualistic perspective and address immediate interpersonal relationships only, which can be either a partner or someone else. The findings suggest that definitions guide the measurements and operationalization of studies. For this reason, we recommend that future definitions be more explicit in recognizing that reproductive autonomy is both an individual and a relational concept and that a person's reproductive autonomy may differ depending on the relationship. Thus, it is important to explicate the influence of a partner, other immediate‐ and distant‐level interpersonal relations, and the wider contextual level. Tosam (2020) has argued that women's reproductive autonomy in communal contexts such as sub‐Saharan Africa should be understood from a relational rather than an individual autonomy perspective because communities may consider women's fertility a communal responsibility. This perspective supports the need to more explicitly include and distinguish the influence of interpersonal relations and contextual dynamics in reproductive autonomy measurements in sub‐Saharan Africa. Distinguishing these various dynamics has already been initiated by Loll et al. (2020, 2021) and suggested by Upadhyay et al. (2014) for the implementation of the reproductive autonomy scale in contexts outside of the USA.

Regarding the measurement of reproductive autonomy, we noticed a lack of available data. Only one study used the open‐access Demographic and Health Survey (DHS) data. However, this study did not measure reproductive autonomy in its entirety; it focused more narrowly on partner dynamics with regard to fertility intentions (Derose and Ezeh 2005). Unfortunately, the DHS does not provide the data required to measure reproductive autonomy, and it predominantly offers data on women of reproductive age. Similarly, Horne, Nii‐Amoo Dodoo, and Naa (2013) indicate that survey data on bridewealth and other norms constraining reproductive autonomy in the African region are lacking. The lack of this type of data is problematic because the findings show that more research is needed to understand the meaning of the individualistic concept of reproductive autonomy in communal settings such as sub‐Saharan Africa as well as to understand the interpersonal and contextual forces that suppress the reproductive autonomy of women and other vulnerable populations. To achieve these insights, we argue that more scholars in sub‐Saharan Africa need to have access to funding and data that enable them to study this topic and provide emic understandings, especially focusing on currently understudied populations and regions.

In relation to the inclusion of subpopulations, we observed various understudied populations. First, we noticed a gap concerning the reproductive autonomy of vulnerable and marginalized groups, such as sexual and gender minorities, migrants, sex workers, and people with disabilities. Sociocultural norms, laws prohibiting same‐sex relationships, and a lack of data may complicate the identification of study participants and the assurance of their safety when conducting research on these populations, although there are examples such as Nyanzi's (2013) study on homosexuality in Uganda. Second, we noticed a gap concerning the reproductive autonomy of men and partners in intimate relationships. As a human right in itself, but also to improve the reproductive autonomy of women, more understanding is needed regarding the reproductive autonomy of men individually and in relation to their partners (Powis 2022; Gottert et al. 2024). The paucity of research studying intimate partners jointly may be explained by a lack of methodology to study them together in a safe way (Obare et al. 2022; Princewill, De Clercq, et al. 2017). For this reason, we recommend developing more methodologies to safely study vulnerable and marginalized populations as well as the power dynamics between intimate partners in both monogamous and polygynous relationships.

With regard to the inclusion of gender and other power dynamics, most publications in this review focused on women's reproductive autonomy either as an outcome in itself or as associated with their reproductive outcomes. This focus seems consistent with the availability of data guiding research and with political agendas and funding agencies prioritizing the empowerment of women and girls (UNFPA 2024; Bill & Melinda Gates Foundation 2024). Subsequently, such research findings may then inform programs and interventions to increase women's reproductive autonomy. However, the findings suggest that such efforts may meet with resistance from husbands, family members, and community members at large, especially when bridewealth has been paid (Nii‐Amoo Dodoo, Horne, and Biney 2014). To illustrate, publications found that financial autonomy may not necessarily result in reproductive autonomy for women and that education for men may be more influential for women's reproductive autonomy and fertility intentions than education for women themselves (Derose and Ezeh 2005; Princewill, Jegede, et al. 2017; Dodoo et al. 2019). These are interesting findings because, during the selection process of our review, we encountered many articles that focused on the influence of financial or decision‐making autonomy on SRHR outcomes.

The findings of our review suggest that research and solutions aimed at enhancing the reproductive autonomy of women and girls must be broadened to better understand how reproductive autonomy might be improved by concentrating on the power dynamics and sociocultural context that both shape and control it. Including the sociocultural context has also been recommended by various publications in this scoping review (e.g., Loll et al. 2019; Kunesh et al. 2024; Decker et al. 2021). For example, future research could investigate the possibilities of gender‐transformative approaches (Njuki et al. 2023; Osuafor and Okoli 2021).

## CONCLUSION

This scoping review has provided various insights and identified gaps in research on reproductive autonomy in sub‐Saharan Africa. In line with a reproductive justice perspective, more research and interventions should focus on the reproductive autonomy of understudied populations and on the interpersonal and contextual forces that counteract initiatives to increase women's reproductive autonomy. Further research is also required to determine the best approaches for safely studying the reproductive autonomy of vulnerable and marginalized populations and the power dynamics between intimate partners, as well as how an individualistic notion like reproductive autonomy might be operationalized in communal contexts. Reproductive autonomy and related concepts, such as reproductive empowerment and reproductive agency, are often used interchangeably, but they differ in scope and meaning. To provide further clarity, a systematic literature review could investigate how the application of these different concepts compares in terms of the insights they provide on the power or freedom to decide and act upon reproductive matters.

## Supporting information



Appendix: Supplemental Materials A1
Appendix: Supplemental Materials A2

## Note

1 Munn et al. (2018) distinguish the following purposes for conducting a scoping review: (1) to identify the types of available evidence in a given field, (2) to clarify key concepts/definitions in the literature, (3) to examine how research is conducted on a certain topic or field, (4) to identify key characteristics or factors related to a concept, (5) as a precursor to a systematic review, and (6) to identify and analyze knowledge gaps. The present study has the second purpose, that is, to clarify key concepts/definitions in the literature.
