# Supplementary material for: Reproductive Autonomy in Fertility Research in Sub‐Saharan Africa: A Scoping Review
Source: Stud Fam Plann. 2025 May 5;56(2):243–73. doi: 10.1111/sifp.70012 (PMC12205730; doi:10.1111/sifp.70012)
Supplement: Supplementary file 1 — Appendix: Supplemental Materials A1 Appendix: Supplemental Materials A2 [file SIFP-56-243-s001.docx]

# Appendix: Supplemental Materials A1

## Search strategy

## Scopus

Search conducted on May 2, 2024.

| **Search** | **Query** | **Records retrieved** |
| --- | --- | --- |
| #1 | “reproductive autonomy” | 2,609 |
| #2 | ((Africa South of the Sahara) OR (Africa south of the Sahara) OR (sub-Saharan) OR (subSaharan) OR (Angola) OR (Benin) OR (Botswana) OR (British Indian Ocean Territory) OR (Burkina Faso) OR (Burundi) OR (Cape Verde) OR (Cabo Verde) OR (Cameroon) OR (Central African Republic) OR (Chad) OR (Comoros) OR (Congo) OR (Cote d'Ivoire) OR (Côte d’Ivoire) OR (Democratic Republic of the Congo) OR (DRC) OR (Zaire) OR (Djibouti) OR (Equatorial Guinea) OR (Eritrea) OR (Eswatini) OR (Swaziland) OR (Ethiopia) OR (French Southern Territories) OR (Gabon) OR (Gambia) OR (Ghana) OR (Guinea NOT guinea pig*) OR (Guinea-Bissau) OR (Kenya) OR (Lesotho) OR (Liberia) OR (Madagascar) OR (Malawi) OR (Mali) OR (Mauritania) OR (Mauritius) OR (Mayotte) OR (Mozambique) OR (Namibia) OR (Niger) OR (Nigeria) OR (Reunion) OR (Réunion) OR (Rwanda) OR (Saint Helena) OR (Sao Tome and Principe) OR (Senegal) OR (Seychelles) OR (Sierra Leone) OR (Somalia) OR (South Africa) OR (South Sudan) OR (Togo) OR (Uganda) OR (Tanzania) OR (United Republic of Tanzania) OR (Zambia) OR (Zimbabwe) OR (Central Africa*) OR (Eastern Africa*) OR (East Africa*) OR (Southern Africa*) OR (Western Africa*) OR (West Africa*) OR (Middle Africa)) | 5,213,719 |
| #3 | 1 AND 2 | 743 |
| Limited to January 1, 1994 – May 2, 2024 | |  |

# Appendix: Supplemental Materials A2

## Data extraction instrument

| **Key information** | **Extracted data** |
| --- | --- |
| Authors |  |
| Title |  |
| Year of publication |  |
| Journal |  |
| Article type |  |
| Country origin |  |
| Aims of the study |  |
| Participants/population (including age and gender) |  |
| Sample size |  |
| Methodology |  |
| Key findings 1a. Definition of reproductive autonomy |  |
| Key findings 1b. Operationalization of reproductive autonomy |  |
| Key findings 2. Subpopulations included at the individual and couple level |  |
| Key findings 3. Inclusion of gender and other power dynamics internal and external to couples |  |
| Notes |  |
